# Detecting Event-Related Spectral Perturbations in Right-Handed Sensorimotor Cortical Responses Using OPM-MEG

**DOI:** 10.3390/bioengineering12101022

**Published:** 2025-09-25

**Authors:** Hao Lu, Yong Li, Min Xiang, Yuyu Ma, Yang Gao, Xiaolin Ning

**Affiliations:** 1 Key Laboratory of Ultra-Weak Magnetic Field Measurement Technology, Ministry of Education, School of Instrumentation and Optoelectronic Engineering, Beihang University, 37 Xueyuan Rd., Haidian Dist., Beijing 100083, China; luhao66@buaa.edu.cn (H.L.); xiang_min@buaa.edu.cn (M.X.); ma_yuyu@buaa.edu.cn (Y.M.); yanggao@buaa.edu.cn (Y.G.); 2 Hangzhou Institute of National Extremely-Weak Magnetic Field Infrastructure, 465, Binan Rd., Binjiang Dist., Hangzhou 310000, China; 3 Hefei National Laboratory, Gaoxin Dist., Hefei 230088, China; 4 Shandong Key Laboratory for Magnetic Field-Free Medicine & Functional Imaging, Institute of Magnetic Field-Free Medicine & Functional Imaging, Shandong University, 27 South Shanda Rd., Licheng Dist., Jinan 250100, China

**Keywords:** OPM-MEG, ERSP, finger movements, motor execution

## Abstract

The optically pumped magnetometer, OPM-MEG, has the potential to replace the traditional low-temperature superconducting quantum interference device, SQUID-MEG. Event-related spectral perturbations (ERSPs) can be used to examine the temporal- and frequency-domain characteristics of a signal. In this paper, a finger-tapping movement paradigm based on auditory cues is adopted, and OPM-MEG is used to measure the functional signals of the brain. The event-related spectral perturbation values of the right and left hands of right-handed people were calculated and compared. The results showed that there was a significant difference in the event-related spectral perturbations between the right and left hands of right-handed people. In summary, OPM-MEG has the ability to measure the event-related spectral perturbations of the brain during finger movements and verify the asymmetry of motor skills.

## 1. Introduction

OPM-MEG [1] is a non-invasive technique for assessing brain function that can record magnetic signals converted from the electrical activity of cortical neurons with millimeter-level spatial resolution and millisecond-level temporal resolution. Traditional SQUID-MEG requires extremely low liquid helium temperatures and thick and bulky Dewar flasks [2]. In recent years, OPM-MEG has been widely used to measure the brain’s magnetic fields. Its specific sensor layout can be more flexibly arranged in the target area of interest, thereby achieving more accurate measurements.

In recent years, optically pumped magnetometers (OPMs) have transformed the landscape of magnetoencephalography (MEG) by offering a cryogen-free, lightweight, and flexible alternative to traditional SQUID-based MEG systems. Alem et al. developed a 128-channel full-head OPM-MEG system with zero-field sensors, achieving high-density coverage of the scalp while enhancing flexibility and integration for neuroimaging applications [3]. However, the system still exhibits limitations in portability and lacks extensive task-based validation. Similarly, Brookes et al. proposed a theoretical framework demonstrating the advantages of triaxial OPM-MEG systems, which improves spatial precision by capturing the full vectorial magnetic field [4]. While promising, their work remains largely theoretical and awaits broader empirical validation.

Several studies have highlighted the improved temporal and spatial resolution of OPM-MEG in task-related paradigms. For example, Gialopsou et al. showed that visually evoked fields recorded with OPM-MEG exhibited clearer spatiotemporal patterns than those from conventional systems, underscoring the system’s precision in early visual areas [5]. An et al. verified the ability of OPM-MEG to detect 40Hz auditory steady-state responses, affirming its sensitivity to higher-frequency activity [6]. Iivanainen et al. extended this further by demonstrating that single-trial classification of auditory-evoked responses using OPM-MEG is comparable to SQUID-MEG, confirming its viability for trial-level analyses [7]. However, these studies predominantly focused on sensory processing tasks and did not examine motor cortex oscillatory dynamics and have not explored hand-dominance effects.

OPM-MEG’s spatial specificity has also been examined in multimodal contexts. An et al. compared the source localization results of OPM-MEG and fMRI in sensorimotor cortex activation, finding that the combination improved localization accuracy [8]. Although this work strengthens the case for OPM-MEG in motor tasks, it did not explore dynamic oscillatory activity such as ERD or ERS during active movement. Likewise, Cho et al. compared resting-state networks between EEG and MEG and confirmed the superior spatiotemporal mapping capabilities of MEG, especially in dynamic network tracking [9]. However, this work was restricted to the resting state and did not involve motor activation or lateralization comparisons.

Some of the most recent literature has begun exploring motor-related oscillatory phenomena. Xu et al. analyzed the temporal–spatial characteristics of beta rebound induced by finger tapping, showing the feasibility of OPM-MEG in capturing task-related beta band activity [10,11,12]. However, gamma band responses and lateralized effects were not thoroughly investigated. Brickwedde et al. and Brookes et al. emphasized the clinical and translational potential of OPM-MEG, especially for pediatric and mobile use cases [13,14]. Yet, both reviews identified gaps in the current research landscape, including the need for more studies focusing on source-resolved functional asymmetries and dynamic task paradigms.

Despite these advances, a critical gap remains: very few studies have directly investigated whether OPM-MEG can reliably differentiate between dominant and non-dominant hand movements in terms of source-level oscillatory features such as beta and gamma ERD/ERS. This distinction is particularly important for understanding motor lateralization, cortical asymmetry, and the neural basis of handedness. To the best of our knowledge, no existing work has systematically evaluated these features using a full-head OPM-MEG setup under tightly controlled motor task conditions.

When individuals perform or plan specific movements, the movement-related areas of the brain often show inhibition and enhancement of neural activity. This regulatory process can be characterized by changes in neural oscillations. As an emerging high-sensitivity neuroimaging technology, OPM-MEG has been successfully applied for the detection of frequency-band responses and theta rhythms in finger abduction and working memory tasks, showing its potential in the study of dynamic neural oscillations. Event-related spectral perturbations (ERSPs) reflect the characteristic changes in neural oscillations in the time–frequency domain, among which event-related desynchronization (ERD) and synchronization (ERS) are important indicators, representing the reduction or enhancement in power in specific frequency bands, respectively. Previous studies have shown that ERD and ERS are widely involved in the planning, execution, and imagination of movements [15,16,17,18,19,20,21], especially in the beta band (13–30 Hz) with richer physiological information [22,23,24,25,26,27,28]. These characteristics not only provide important clues for the study of movement mechanisms but also show application prospects in the diagnosis and rehabilitation assessment of movement disorders [29,30,31] and are expected to become potential biomarkers for movement decoding. However, systematic characterization of ERD/ERS phenomena in bilateral finger movement tasks using OPM-MEG remains limited. Therefore, clarifying the ability of OPM-MEG to characterize the regulation of neural oscillations in such motor tasks will help promote its in-depth application in the field of motor neuroscience.

This study conducted a real finger-tapping experiment, calculated the ERSP value, and compared it with the results of the right and left hands. This paper aims to verify the ability of OPM-MEG to measure the difference between the right and left hands of right-handed people.

## 2. Materials and Methods

### 2.1. Experimental Subjects

Seven healthy subjects (6 males and 1 female) participated in this study. The subjects were aged 24–29 years old, native Chinese speakers, right-handed, and had no congenital developmental diseases, hearing impairment, or neurological or psychiatric disorders. All subjects signed an informed consent form and agreed to participate in this study. The study protocol was in accordance with the Declaration of Helsinki and was approved by the Ethics Committee of Beihang University.

### 2.2. OPM-MEG System

**Figure 1:**
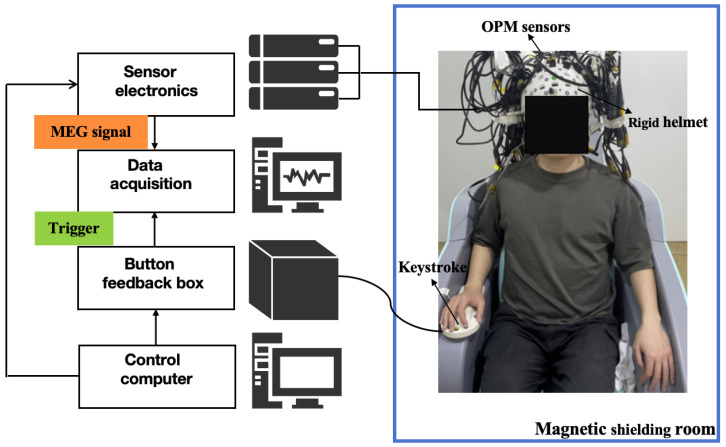
OPM-MEG system.

In this study, a multi-channel OPM-MEG system was constructed using a 32-channel OPM sensor (QZFM, QuSpin, Louisville, CO, USA) to measure the radial component of the magnetic field. The schematic diagram of the system is shown in Figure 1. During the experiment, the OPM-MEG system was operated in a magnetic shielding room (MSR), and the subjects sat on a non-magnetic chair in the central area of the room during the entire recording process. The MSR [32,33] consisted of two layers of high-magnetic-permeability alloy and one layer of copper and was equipped with a demagnetization coil [34,35] to reduce the magnetization of the high-magnetic-permeability alloy layer. The remanence in the central area of the MSR was less than 10 nT, and the magnetic field gradient was less than 0.1 nT/cm. For different subjects, the same rigid helmet was used, and the sensors were placed in the same slots. The sensor depth for each subject was manually adjusted to ensure that the OPM sensor was as close to the scalp as possible.

The outputs of all channels were recorded by a digital data acquisition (DAQ) system (ART Technology, Beijing, China), which consisted of an ARTPXI-7685 chassis and a PXI9009 board (ART Technology, Beijing, China) with a sampling frequency of 1 KHz. The auditory stimulation device consisted of a sound card (UMC202HD, Autodesk Electronics Co., Ltd., Zhongshan, Guangdong, China), headphones (10 m ER3C insert earphones, Tinnitus Research, Elk Grove Village, IL, USA), and plastic catheters. The auditory stimulation sequences were generated by Psychology Toolbox software 3.0.18. The sound stimuli were connected to ER3C headphones through a sound card and then connected to the plastic catheters for transmission to the MSR. The finger-press feedback device was provided by Shenzhen Medtech Medical Co., Ltd., Shenzhen, China. The triggering time of the sound stimuli, finger-press feedback, and brain signals were synchronously recorded by the DAQ system. The electrical module of the sensor was placed outside the MSR to avoid electromagnetic interference.

### 2.3. Experimental Design and Preprocessing

**Figure 2:**
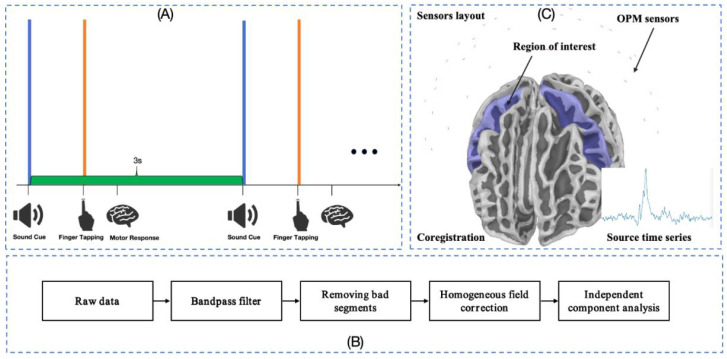
(**A**) Experimental paradigm; (**B**) preprocessing; (**C**) registration.

In order to verify the inter-trial consistency caused by finger tapping movements, a continuous finger-tapping movement paradigm was designed in references [36,37]. The schematic diagram of the experimental paradigm design is shown in Figure 2A. In the experiment, the participants kept their eyes open and were asked to look at a specific target to avoid eye movements. When the auditory stimulus (1000 Hz, 64 dB) was heard but not annoying to the subjects, the subjects immediately tapped quickly with their right index finger. The left-hand task was performed in the same manner and for the same duration as the right-hand task. Before the experiment, participants were trained to tap a key with their right index finger immediately after hearing the auditory cue. A total of 200 trials were designed for each hand, and, after removing artifacts and noise, approximately 180 valid trials per hand were retained for further analysis.

The data analysis process is shown in Figure 2B. First, OPM-MEG data between 2 and 40 Hz were filtered, and bad segments were manually identified and eliminated. Then, homogeneous magnetic field correction [38] was used to reduce interference across the entire spectrum. In addition, independent component analysis was used to eliminate artifacts such as heartbeat, blinking, and muscles. The data were segmented with the tapping action as time zero, and each epoch ranged from 1.0 s before to 2.0 s after the action. The auditory cue was used only to guide the subjects to maintain a steady rhythm and did not serve as a segmentation trigger.

### 2.4. Registration and Source Localization

As shown in Figure 2C, in the OPM-MEG experiments, 3D digital images of the participants’ heads were collected by an optical imaging system (Einscan H, SHINING 3D, Hangzhou, China) and used to align these 3D digital images with the scalp surface extracted from the MRI to obtain the relative position and orientation between the OPM sensor and the MRI, as well as the relative position between the MEG sensor and the MRI. Figure 2 shows the final positions of OPM sensors relative to the brain.

The MRI data of the subjects were acquired by Siemens MAGNETOM Vida 3.0T biomatrix system. The data of all subjects were acquired by T1-weighted MRI scanner using MPRAGE sequence (TR, 2200 ms; TE, 2.53 ms; TI, 1000 ms; FA, 8; field of view, 256 × 256 × 192 mm; voxel size, 1 × 1 × 0.8 mm).

The MRI data were reconstructed using Freesurfer software v7.2.0 [39]. The watershed algorithm was then applied to separate the cerebral cortex, skull, and scalp to obtain a three-layer boundary element model of the subject’s brain. The noise covariance matrix was estimated based on the baseline data before the stimulus (−1–0.0 s). The source distribution was then estimated using the MNE method [40]. The depth weighting was set to 0.8 to compensate for the bias of the minimum norm estimate toward the surface [41].

In this paper, the source space is restricted to the region of interest covered by the sensor (the precentral gyrus (blue) in Figure 2C). To compare source distributions between different stimuli, the cortical surface of each subject was mapped to the “fsaverage” template [42]. These calculations were implemented in MNE-Python [43,44]. The registration kit was provided by [45]

To investigate brain activity measured by OPM-MEG, we determined the locations of regions of interest (ROIs). The ROIs were defined based on the location of the maximum power ratio activity within Brodmann’s BA4 region, which was based on the location where the maximum difference in activity between post- and pre-tap responses was observed.

### 2.5. ERSP

Event-related desynchronization and synchronization (ERD/ERS) are brain oscillation activities in a specific frequency band extracted from magnetoencephalogram signals and have been widely used in motion-related research. ERSP describes the relative change in the MEG power spectrum of a specific event in an experiment and is calculated using wavelets. The event-related spectrum of a single experiment can be estimated as

(1) $$P_{k}=\frac{{\left|F_{k}\left(f,t\right)\right|}^{2}}{\mu_{B}\left(f,k\right)}$$

where $F_{k}\left(f,t\right)$ is the frequency *f* and spectral estimate at time *t* for the *k*-th experiment. $\mu_{B}\left(f,k\right)$ is the average baseline spectral estimate for experiment *k* at frequency *f*, defined as

(2) $$\mu_{B}\left(f,k\right)=\frac{1}{m}\sum_{t^{\prime }\in B}{\left|F_{k}\left(f,t^{\prime }\right)\right|}^{2}$$

where *B* is the set of time points during the baseline period, and *m* is the total number of time points during the baseline period. The ERSP estimate for a single experiment can be calculated as

(3) $$ERSP_{\sin gle}=10log_{10}\left(Pi\right)$$

Considering that MEG signals are non-stationary signals with high variability, in order to reduce the randomness of the signal, by averaging the ERSP values of the subjects, the average event-related spectrum can be estimated as

(4) $$S\left(f,t\right)=\frac{\frac{1}{n}\sum_{i=1}^{n}{\left|F_{k}\left(f,t\right)\right|}^{2}}{\mu {’}_{B}\left(f\right)}$$

where ${\mu^{\prime }}_{B}\left(f\right)$ is the average spectral estimate of all baseline points at frequency *f* and is defined as

(5) $$\mu {’}_{B}\left(f\right)=\frac{1}{mn}\sum_{k=1}^{n}\sum_{t^{\prime }\in B}{\left|F_{k}\left(f,t’\right)\right|}^{2}$$

where *n* is the number of experiments for the average calculation. The average ERSP can be calculated as

(6) $$ERSP_{mean}=10log_{10}\left(S\left(f,t\right)\right)$$

ERS/ERD is calculated using the following formula:

(7) $$ERD/ERS\left(f,t\right)=\frac{P\left(f,t\right)-P_{\mathrm{baseline}}\left(f\right)}{P_{\mathrm{baseline}}\left(f\right)}\times 100\%$$

where P(f,t) represents the spectral power at frequency *f* and time *t* within the post-stimulus window (200–800 ms), $P_{\mathrm{baseline}}\left(f\right)$ is the mean spectral power at frequency *f* during the baseline interval (−1600 to −1000 ms), A negative value indicates ERD (desynchronization), while a positive value indicates ERS (synchronization).

### 2.6. ERD/ERS Quantification

Spectrograms were calculated for frequencies from 2 Hz to 40 Hz. Based on the spectrograms, we selected the most reactive single frequency band in the beta range spanning 2 Hz. ERD or ERS was defined as the percentage power decrease (ERD) or power increase (ERS) relative to the baseline time interval, which in our experiments was −1.6 s to −1 s before stimulus onset. The power average was then calculated based on the wavelet-based estimator for all analyzed frequency bands and wavelet coefficients for different time windows to determine the most reactive frequency band.

### 2.7. Statistical Analysis

In this study, the Wilcoxon signed-rank test [46] was applied at each time point during the stimulation phase to statistically compare the ERSP values of the right-hand task condition and the left-hand task condition. The ERSP value of each subject within 1 s after stimulation under the two conditions was extracted and used as a paired sample. The null hypothesis was that there was no difference in the mean ERSP value between the right-hand task condition and the left-hand task condition. Then, a pseudo t statistic was constructed as the difference between the means of the two conditions and normalized by their pooled variance. The significance level (*p*-value) of the null hypothesis was determined based on the distribution of the pseudo t statistic. To address the problem of multiple comparisons, the false discovery rate (FDR) method [47] was used to correct the *p*-value.

## 3. Results

### 3.1. Analysis of Oscillation Activity of Contralateral Brain

The percentage increase in ERS rebound in the contralateral brain ranged from 10% to 60%. The percentage increase of ERS rebound in the theta band was 12% in the left hand and 10% in the right hand. The percentage increase in ERS rebound in the alpha band was about 10% in both the left and right hands. The percentage increase in ERS rebound in the beta band was about 30% in both the left and right hands, while the percentage increase in ERS rebound in the gamma band was 60% in the left hand and 40% in the right hand. (Statistically significant time frequency points are indicated in white, *p* < 0.005, FDR-corrected.)

The ERD percentage in the contralateral brain decreased between 5% and 40%. The ERD percentage in the theta band decreased by 10% in the left hand and 8% in the right hand; the ERD percentage in the alpha band decreased by about 15% in both the left and right hands; the ERD percentage in the beta band decreased by 40% in the left hand and 35% in the right hand; and the ERD percentage in the gamma band decreased by 20% in the left hand and 15% in the right hand. (The time–frequency points with statistical significance are indicated in white, *p* < 0.005, FDR correction.)

**Figure 3:**
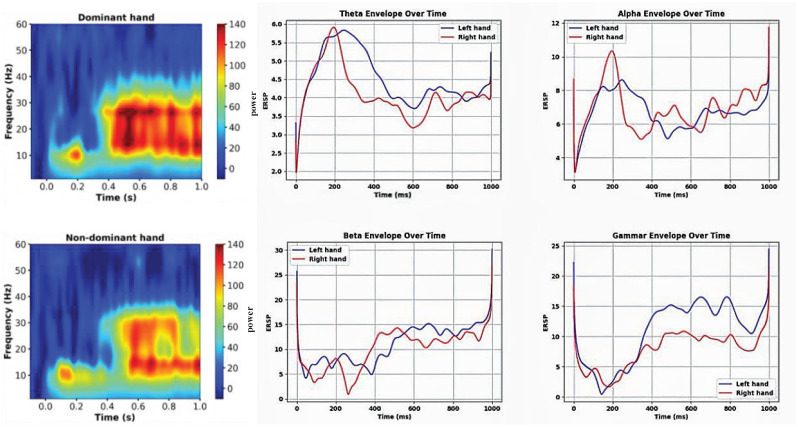
Event-related desynchronization/synchronization (ERD/ERS) in the contralateral primary motor cortex.

As shown in Figure 3, the percentage increase in ERS rebound in the contralateral brain ranged from 8% to 60%. The percentage increase in ERS rebound in theta band was about 15% in both the left and right hands. The percentage increase in ERS rebound in the alpha band was 30% in the left hand and 20% in the right hand. The percentage increase in ERS rebound in the beta band was 50% in the left hand and 20% in the right hand, and the percentage increase in ERS rebound in the gamma band was 60% in the left hand and 10% in the right hand. (The statistically significant time–frequency points are indicated in white, *p* < 0.005, FDR-corrected.)

### 3.2. Analysis of Oscillation Activity of Ipsilateral Brain

**Figure 4:**
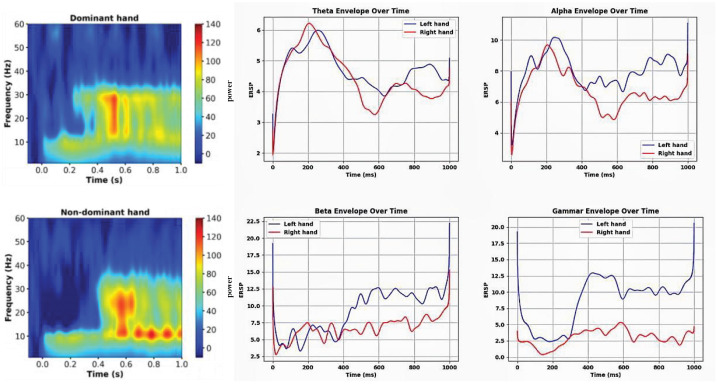
Event-related desynchronization/synchronization (ERD/ERS) in the ipsilateral primary motor cortex.

As shown in Figure 4, the percentage reduction in ipsilateral ERD ranged from 5% to 45%. The percentage reduction in ERD in the theta band was approximately 10% in both the left and right hands; the percentage reduction in ERD in the alpha band was 25% in the left hand and 20% in the right hand; the percentage reduction in ERD in the beta band was 45% in the left hand and 30% in the right hand; and the percentage reduction in ERD in the gamma band was 15% in the left hand and 10% in the right hand. (The time–frequency points with statistical significance are indicated in white, *p* < 0.005, FDR correction.)

Compared to previous OPM-MEG studies that focused on either specific motor tasks or selected frequency bands, our study provides a more comprehensive and symmetric characterization of oscillatory responses in both hemispheres during left- and right-hand finger tapping. We quantified the event-related synchronization (ERS) rebound across four frequency bands (theta, alpha, beta, and gamma), revealing consistent contralateral activations and distinct lateralized responses in the ipsilateral cortex. Notably, the ipsilateral beta and gamma ERS rebound after left-hand tapping reached up to 60%, substantially higher than reported in previous studies focusing on unilateral movements. Moreover, this hemispheric dissociation was captured using a single modality (OPM-MEG), demonstrating the system’s reliability for bilaterally balanced motor assessments.

## 4. Discussion

The contralateral cortex showed a consistent pattern of strong ERD-ERS shifts in all frequency bands, especially in the beta and gamma bands, reflecting its core role in motor control, execution, and feedback regulation. The activity of the ipsilateral cortex showed greater task and frequency band dependence, especially in the left-hand task (for the right cortex), revealing that more cortical resources are required in the control process of the non-dominant hand.

OPM-MEG source-level time-0frequency analysis showed that beta-band activity within the sensorimotor cortex decreased in all subjects during finger tapping, followed by a rebound, as previously reported during index finger abduction or tapping. This study used epoch segmentation with the tapping action as the trigger rather than the auditory stimulus as the zero time to more directly reflect the neural activity associated with movement execution. To the best of the authors’ knowledge, this is the first study to measure oscillatory features between the left and right hands of right-handed individuals in a motor paradigm using OPM sensor signals, which helps demonstrate the power of OPM-MEG in characterizing functional connectivity in cortical networks.

The parietal lobe is an important cortex in the processing of motor information. In particular, the beta and gamma bands also reflect motor perception and decision making. In this study, we designed a finger-tapping task to explore oscillatory activities across frequency bands using OPM-MEG. Consistent with previous findings, the contralateral cortex showed stronger ERS rebound than the ipsilateral cortex, particularly in the beta and gamma bands. Importantly, the ipsilateral cortex exhibited clear asymmetry: ERS rebound was more pronounced during left-hand movements (non-dominant hand) than during right-hand movements (dominant hand). This hemispheric dissociation suggests that additional cortical resources are recruited when controlling the non-dominant hand. Beta rhythm represents motor cortical activity, including movement planning and execution [48,49,50,51]. Therefore, the synchronization/desynchronization characteristics of beta rhythm are expected to play an important role in detecting movement intention in people with physical disabilities. Due to experimental limitations, left-handed participants were not included in the present study but could be examined in future work.

Our results extend the existing body of the OPM-MEG literature in several key ways. While earlier studies such as Xu et al. (2025) [10] and Gialopsou et al. (2021) [5] validated the temporal–spatial sensitivity of OPM-MEG in motor and sensory domains, they often limited their analyses to contralateral responses or specific frequency ranges (e.g., beta band only). In contrast, our study reveals both bilateral and multi-frequency ERS dynamics during rhythmic motor execution, providing a richer depiction of cortical oscillatory behavior. Additionally, our use of OPM-MEG to segment epochs based on motor performance rather than auditory cues offers a more behaviorally anchored view of brain dynamics. This methodological shift addresses concerns raised in earlier works [52,53] about the reliance on external triggers, enhancing ecological validity and temporal alignment between neural events and behavioral output. The asymmetric ERS rebound observed in the ipsilateral cortex, particularly in the gamma band (left hand > right hand), may reflect hemispheric differences in interhemispheric inhibition or cortical compensation mechanisms—an aspect rarely reported with such clarity in OPM studies. Our findings thus align with and go beyond recent reports on lateralized motor responses in traditional MEG and fMRI studies [54], demonstrating that OPM-MEG can robustly capture subtle hemispheric dynamics.

## 5. Conclusions

This paper uses the OPM-MEG system to measure right-hand and left-hand finger tapping. The feasibility and accuracy of OPM-MEG measurement of ERSP under the motion paradigm were evaluated. Both the left and right hands exhibited typical beta-band ERD/ERS patterns but with significant asymmetries in hemispheric distribution. The results demonstrate the feasibility and reliability of OPM-MEG in measuring ERSPs during a finger movement task. While the core objective of this study was not a complex cognitive study, it aimed to verify the ability of OPM-MEG to detect ERSPs in an auditory-cued rhythmic finger-tapping paradigm. This may inform future clinical applications in populations such as those with stroke or Parkinson’s disease.

## Data Availability

The data, aside from the data published in this manuscript, are not publicly available due to privacy restrictions.
